# Metabolism under hypoxia in Tm1 murine melanoma cells is affected by the presence of galectin-3, a metabolomics approach

**DOI:** 10.1186/2193-1801-3-470

**Published:** 2014-08-26

**Authors:** Pedro Starzynski Bacchi, Antonio Carlos Bloise, Silvina Odete Bustos, Lara Zimmermann, Roger Chammas, Said Rahnamaye Rabbani

**Affiliations:** Laboratório de Oncologia Experimental and Centro de Investigação Translacional em Oncologia, Instituto do Câncer do Estado de São Paulo and Faculdade de Medicina da Universidade de São Paulo, Av. Dr. Arnaldo, 251, CEP 01246-000 São Paulo, Brazil; Laboratório de Ressonância Magnética, Instituto de Física da Universidade de São Paulo, R. do Matão trav. R, 187, CEP 05508-090 São Paulo, Brazil

**Keywords:** *Metabolomics*, Galectin-3, Melanoma, ^1^H-MRS, NMR, PCA, Warburg effect, *Tm1.G2*, *Tm1.N3*

## Abstract

*Metabolomics* has proven an useful tool for systems biology. Here we have used a *metabolomics* approach to identify conditions in which *de novo* expression of an established tumor marker, galectin-3, would confer a potential selective advantage for melanoma growth and survival. A murine melanoma cell line (*Tm1*) that lacks galectin-3 was modified to express it or not (*Tm1.G2* and *Tm1.N3*, respectively). These variant cell line were then exposed to conditions of controlled oxygen tensions and glucose levels. Metabolic profiling of intracellular metabolites of cells exposed to these conditions was obtained in steady state using high resolution ^1^H Magnetic Resonance Spectroscopy (^1^H-MRS) and multivariate statistical analysis. The Nuclear Magnetic Resonance (NMR) spectra contained a large number of absorption lines from which we were able to distinguish 20 metabolites, 3 fatty acids and some absorption lines and clusters were not identified. Principal Components Analysis (PCA) allowed for the discrimination of 2 experimental conditions in which expression of the tumor marker galectin-3 may play a significant role, namely exposure of cells to hypoxia under high glucose. Interestingly, under all other experimental conditions tested, the cellular system was quite robust. Our results suggest that the *Metabolomics* approach can be used to access information about changes in many metabolic pathways induced in tumorigenic cells and to allow the evaluation of their behavior in controlled environmental conditions or selective pressures.

## Background

Melanomas are tumors derived from melanocytes, the pigment-producing cells of the skin (Velho, 2012). Cells that undergo the carcinogenic process, i.e. transform into tumor cells, present intracellular changes, such as immortalization, independence of growth signals and metabolic changes that significantly modify the intracellular and the tumor microenvironment (Hanahan and Weinberg, 2011). One of these changes was first described by Otto Warburg, who observed modifications in the metabolism of intracellular glucose. Warburg observed a curious effect, named after him, in which the tumor cells were characteristically glycolytic even at normal oxygen concentrations. The Warburg effect appears disadvantageous because glycolysis generates 9 times less ATP than oxidative phosphorylation, in addition, the production of lactate generates an exacerbated acidosis in the tumor microenvironment that is harmful to normal cells; furthermore, cells begin to import larger amount of glucose that can be diverted for PPP (pentosephosphate) pathway, which produces NADPH and ribose-5-phosphate (Heiden et al. 2009).

Apparently the Warburg effect makes the transformed cell more resistant. Since intratumoral blood flow is very heterogeneous and oxygen concentrations vary widely, cells that depend on oxidative phosphorylation are impaired within tumors. The occurrence of the Warburg effect is associated with activation of oncogenes such as Akt and Myc and has a close connection with the hypoxia-induced factor (HIF-1), which is involved in the induction of glycolysis (Gatenby and Gillies, 2004; Heiden et al. 2009).

A model of murine melanoma progression was derived from a murine melanocyte cell line (*Melan a*) through repeated cycles of cell deadhesion (Oba-Shinjo et al. 2006). This model included several cell lines, such as *Tm5* and *Tm1*, the latter used in the present work. Previous studies indicated that these cell lines had a down regulation of proteins involved in ROS degradation and were more pro-oxidative than the parental cell line, *Melan a*, and survived in conditions of oxidative stress (de Souza et al. 2006). Transcriptomic analysis of malignant transformation indicated that one of the major differences between *Tm1* cells and the parental cell line was the loss of expression of *gal-3* in the tumorigenic cell line, *Tm1* (de Souza et al. 2006).

Galectin-3 is a multifunctional protein member of the group of lectins that bind to β-galactosides. *Gal-3* is involved in different biological processes and these functions are dependent of its subcellular localization. In the extracellular space the biological activities of *gal-3* involve interactions with galactosides, useful to mediate cell-cell adhesion and modulate negatively the cell adhesion to ECM proteins (Nangia-Makker et al. 2008). *Gal-3* can also cross-link glycoconjugates present on the cell surface in order to activate several signaling pathways associated with cell death and apoptosis (Newlaczyl and Yu, 2011). *Gal-3* is capable to bind various molecules in the cells; which could explain its contrasting functions. In the intracellular environment, *gal-3* is involved in differentiation, regulation of cell proliferation through interactions with K-Ras (Levy et al. 2010) or with phosphatidylinositide 3-kinases (PI3K-Akt), and cell survival. In the cytoplasm, galectin-3 inhibits *cytochrome c* release from mitochondria, thus preventing caspase-3 activation, possibly through an interaction with the anti-apoptotic protein bcl-2. Also, *gal-3* is enriched in mitochondria and it seems to regulate the mitochondrial homeostasis, acting as an apoptotic inhibitor (Matarrese et al. 2000). *Gal-3* interacts physically with the mitochondrial ATP synthase among other proteins (Carvalho et al. 2014) and decrease mitochondrial ATP production in cancer cells (Kim et al. 2008). Inside the nucleus, *gal-3* promotes the pre-mRNA splicing and can enhance or stabilize transcription factors. Furthermore, *gal-3* has an important role in transduction of Wnt/beta-catenin pathway, related with development, tissue homeostasis and tumor growth. In addition, *gal-3* directly activates AP-1, a transcription factor that transcribes metalloproteinases such as MMP-1, involved in invasion (Dye et al. 2013).

Melanocytes residing in the basal layer of the epidermis are in relatively hypoxic microenvironment. It is documented that melanoma development and aggressiveness can be modulated by various tumor microenvironmental factors including underoxygenation of the tumor mass (Bedogni and Powell, 2009). At the same time, *gal-3* expression is responsive to hypoxia and seems to be regulated by HIF-1α (Zeng et al. 2007). In order to establish the possible role of *gal-3* in murine melanomas, we had then transfected *Tm1* cells with either an empty vector (*Tm1.N3*) or a vector coding *gal-3* gene (*Tm1.G2*). Thus, a controlled cellular system was generated to determining the impact of *gal-3* expression in mitochondrial metabolism of tumorigenic cells and a *metabolomics* approach was used to map quantitatively the intermediates of glucose metabolism by measuring their concentrations in distinct cell lines at varying concentrations of oxygen and glucose.

The *Metabolomics* approach results from spectroscopy methods such as Raman, Infra-Red (FT-IR), mass spectrometry (liquid and gas) and ^1^H MRS combined with multivariate statistical analysis (Griffin, 2004). This method, for example, can be applied as diagnostic tool to study changes in metabolic pathways induced by a certain pathological situation such as melanomas (Triba et al. 2010) or *mdx*, animal model for Duchenne Muscular Dystrophy (Martins-Bach et al. 2012). In this work high resolution NMR spectra were used to study the changes in concentration levels of each metabolite and through multivariate statistical analysis (*Principal Component Analysis* – PCA), patterns were identified and associated with varying conditions of tumor cell exposure. A fingerprint was then obtained from either galectin-3 positive or negative cells under different metabolic conditions.

## Materials and methods

### Cell culture

**Figure 1:**
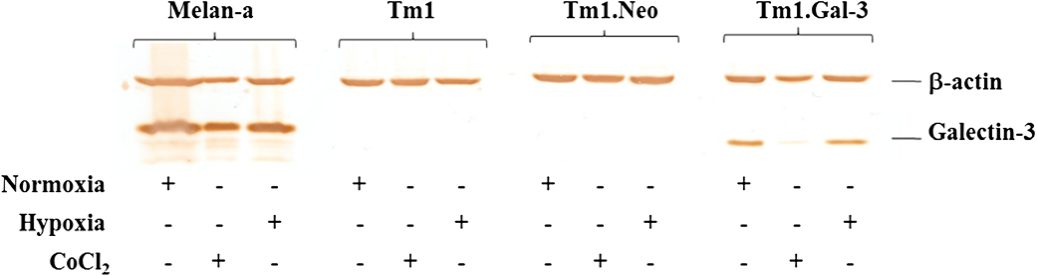
**Galectin-3 accumulation did not alter upon exposure of cells to hypoxic conditions.** The normal murine melanocyte cell line, *Melan-a*, gave rise to the melanoma cell line *Tm1*, as described elsewhere (Oba-Shinjo et al. 2006). Lack of galectin-3 expression was among the differences between the tumor cell lines and their normal counterpart (de Souza et al. 2006). We had then transfected the galectin-3 gene in the tumorigenic cell line *Tm1*, obtaining clones such as the ones used in this study. Control transfectants were obtained by transfection of the empty plasmid pEF1.neo (*Tm1.Neo* cells, *Tm1.Nx* cells) and the galectin-3 expressing cell was obtained by transfection of the galectin-3 coding plasmid pEF1.neo/gal3 (*Tm1.Gal-3*, *Tm1.Gx* cells). Protein extracts of the cell lines studied were routinely analyzed by western blots. Loading controls were done analyzing β-actin accumulation and galectin-3 expression was analyzed with specific monoclonal antibodies (M3/38, rat anti-galectin-3). Exposure of cells to hypoxia (atmospheres containing 1% oxygen) for 24 hours did not interfere with accumulation of galectin-3, differently from the exposure of cells to cobaltous chloride, a prolyl-hydroxylase inhibitor (and so-called “hypoxic mimetic” drug). Western blots were done after resolving protein extracts in 10% SDS-polyacrylamide gels. Plus and minus signs represent whether cells were exposed or not to a given experimental condition.

Murine cells were cultured in RPMI-1640 medium, pH adjusted to 7.2 and supplemented with 5% of a mixture consisting of fetal bovine serum as a supplement to stimulate cell division. From the tumorigenic cell line *Tm1*, two new variants were obtained by cell transfection procedures in order to obtain *Tm1.G2* (cells that restart to express *gal-3* due to a specific transfection) and *Tm1.N3* (cells that remain not expressing the *gal-3* after the transfection was done by a plasmid; also called control transfection) (Figure 1). G418 antibiotic was added in the cultures of transfected cells. To detach cells, the entire medium was aspirated through a Pasteur pipette attached to the vacuum pump. The cells were washed twice with PBS/EDTA; trypsin was finally added and its activity inhibited thereafter by addition of supplemented medium. *Tm1.G2* and *Tm1.N3* were submitted to two different conditions of oxygen tension (hypoxia, 1% oxygen gas concentration and normoxia with 20% of oxygen concentration) and glucose levels (high *–* 25.0 mM and control *–* 11.1 mM). Previous analyses showed that exposure of cells to hypoxia, as defined above, did not alter *gal-3* expression in the cell variants studied herein (Figure 1). Approximately, 1 × 10^7^ cells were counted for each cell line using trypan blue staining (for exclusion of non-viable cells) in a Neubauer chamber viewed with light microscopy. Hypoxia condition was achieved by using an appropriate chamber (Modular Incubator Chamber, Billups-Rothenberg) filled with 95% of nitrogen and 5% of carbon dioxide. After 10 minutes of gases exchange, the chamber was sealed and placed in a humidified atmosphere at 37°C for 24 hours and oxygen concentration monitored by an oxymeter (Dräger – PAC 3000).

### NMR sample preparation

Cells collected upon 90% confluency and > 95% viable, as indicated by trypan blue exclusion staining were separated for NMR analysis. Cells were homogenized in 250 μL of methanol/water (1:1) solution at 4°C (Wu et al. 2008), sonicated 5 times for a period of 1 minute with intervals of 10 seconds between rounds of sonication. The reagents and cells were mixed with a rapid manual shaking and centrifuged at 13000 *g* for 20 minutes. Finally, supernatant (methanol, water and metabolites) was collected and lyophilized. The lyophilized extracts were diluted in 600 μL of deuterium oxide (D_2_O, Sigma-Aldrich) and transferred into 5 mm resonance tubes. Capillaries containing ~ 2 μL a solution of D_2_O and 0.75% 3-(trimethylsilyl) propionic*-2,2,3,3-d*_4_ acid, sodium salt (TSP, Sigma-Aldrich) were inserted in the tubes to be used as NMR reference. Tubes were then stored at 4°C until the beginning of resonance experiments. The combination of the 3 preparation conditions mentioned before as cell transfection (*G2* and *N3*), oxygen tension (hypoxia and normoxia) and glucose levels (high and control) resulted in 8 combinations (groups). Besides, each combination also contained 3 independent samples (triplicate) in order to aggregate statistical value to the results, which results in 24 samples analyzed by NMR.

### NMR spectroscopy

High resolution ^1^H NMR spectra were acquired in a Varian spectrometer (Varian Associated, Inc., Palo Alto, CA, USA) operating at 9.4 T, corresponding to 400 MHz for protons, using a 5 mm multinuclear probe with 3 channels: decoupling, observe and deuterium lock. Spectra were acquired at 21°C using the single pulse sequence with following parameters: relaxation delay 6.0 s, excitation pulse width 3.7 μs, acquisition time 4.1 s, spectral window 4.0 kHz, 32Kb data points and 1024 transients (approximately, 3 hours *per* experiment). Selective saturation of residual water was carried out using pulse width of 1.5 s at water position. A weighted Fourier transform with an exponential function corresponding to 0.3 Hz line broadening was applied to the spectra followed by phase and baseline correction. The region from 4.50 to 5.50 ppm of spectra, overlapping the water absorption, was not considered in analysis and the vertical scale was normalized by the TSP area integrated over -0.008 to 0.008 ppm range. Subsequently, the spectra were also normalized by its total area integrated over 0.45 to 4.50 ppm range (aliphatic hydrogens).

### Statistics and pattern recognition processing of data

The normalized absorption line/lines corresponding to different metabolite were integrated over the extension of the peak/peaks and each integral was used as one input to the multivariate matrix. In general, the metabolite identification was done using Chenomx (Chenomx NMR Suite, Chenomx Inc.) (Chenomx, 2011). However, some of this regions (or peaks) could not be appropriately identified by Chenomx and therefore other data base were used, such as, BMRB - *Biological Magnetic Resonance Bank* (Ulrich et al. 2008) and some references (Jansen et al. 2006; Romanska et al. 2009; Shi et al. 2008; Triba et al. 2010). The total area under the spectra was calculated taking in to account the entire spectra (0.45 – 4.5 ppm), while the area of each absorption line was calculated taking in to account only the extension of each line or interval of lines. Therefore, the total sum of the areas from absorption lines is slightly smaller than the total area under the spectra. To make these two areas equal, we had to add an extra absorption line, *P*, with the area equal to total area minus the sum of the areas of the absorption lines.

In order to obtain the relative concentration of the metabolites the average normalized area of each peak *per* triplicate was calculated and arranged in a *multivariate matrix* (8 × 30), 8 groups and 30 peaks, used in statistical analysis. The multivariate statistical methods chosen in this work was PCA (SIMCA-P + 11; Umetrics) (Umetrics, 2007) using auto-scaled analysis, where all input variables’ variances were re-normalized to unit variance. This normalization provides a reliable identification of variation in metabolites with low concentration that normally would not be detectable by naked eyes (Griffin et al. 2001a; Griffin et al. 2001b). When clusters of points were observed in the *score plots*, *loading plots* were analyzed to identify the metabolites which are responsible for the clustering of similar samples. In parallel to the multivariate analysis, the differential metabolites pointed by PCA were validated at a univariate level using ANOVA and Fisher’s test. The critical *α*-value used as threshold in this study was set to 0.05.

## Results

In order to understand the impact of *gal-3* expression in mitochondrial metabolism, spectra of 24 samples, divided in 8 groups, were obtained. Each group contained 3 sets of samples with identical nominal transfection, oxygen tension and subjected to same concentration of glucose. Each sample was specified by three letters: the first letter indicates the cell line, “*G*” for *Tm1.G2* and “*N*” for *Tm1.N3*, the second letter indicates the oxygen tension, “*h*” for hypoxia and “*n*” for normoxia and, finally, the third letter indicates the glucose concentration used in cultures, “*h*” for high and “*c*” for control.

**Figure 2:**
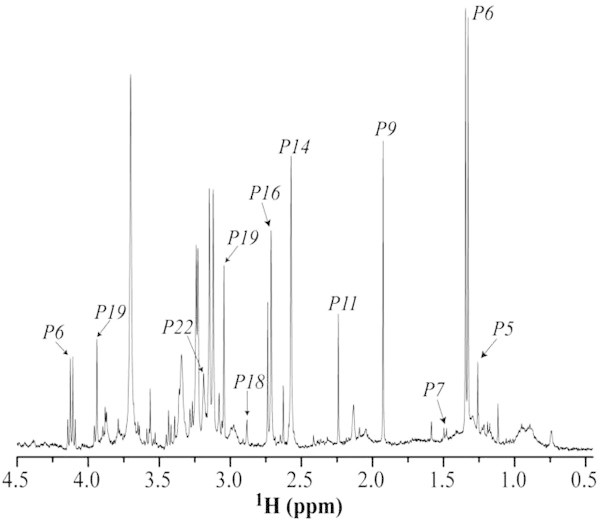
**Typical** ^**1**^ **H high resolution spectrum, obtained at 400 MHz and 21°C, from melanoma cells (** ***Tm1.G2*** **hypoxia and high glucose) showing only the chemical shift range corresponding to aliphatic hydrogen (4.5 – 0.45 ppm).** The absorption lines due to 3-*hydroxyisovalerate* (P5), *lactate* (P6), *alanine* (P7), *acetate* (P9), *acetone* (P11), *methylamine* (P14), *dimethylamine* (P16), *trimethylamine* (P18), *creatine* (P19) and *free choline* (P22) are depicted with arrows.

Figure 2 shows a typical melanoma (*Tm1.G2* hypoxia and high glucose, *Ghh*) ^1^H-MRS spectrum, each one contains a large number of absorption lines from which we were able to distinguish 20 metabolites, 3 fatty acids located at 0.740, ≈ 0.9 and 1.534 ppm, 6 unidentified absorption lines and few other lines, which were not considered, due to their small intensity compared to other absorption lines. As it can be seen in Table 1, the absorption lines corresponding to these 20 metabolites can have a singlet, doublet, triplet, quartet or multiplet structures. For example, lactate is composed by a doublet at 1.329 and 1.346 ppm and a quartet at 4.082 – 4.153 ppm interval. Furthermore, there is an interval (4.180 – 4.470 ppm), which corresponds to the overlap of absorption lines of AMP, ADP, ATP, GDP, NADP^+^ and a single line at 2.134 ppm, which could correspond to different metabolites, see Table 1.

**Table 1:** **Assignments of resonance peaks obtained from** ^**1**^ **H-MRS data**

	***Peaks***	***Metabolites***
1	0.740	Cholesterol C-18 methyl singlet
2	≈0.900	fatty acid chains ω-CH_3_
3	1.115	Unidentified
4	(1.176,1.192)	3-hydroxybutyrate
5	1.260	3-hydroxyisovalerate
6	(1.329,1.346) and (4.082 – 4.153)	Lactate
7	(1.477,1.495) and (3.740 – 3.796)	Alanine
8	1.584	fatty acid β-methylene (CH_2_CH_2_COO)
9	1.924	Acetate
10	2.134	Unidentified (methionine, acetylcholine, glutamate, homocysteine)
11	2.240	Acetone
12	(2.343,2.363,2.381)	Glutamate
13	2.413	Succinate
14	2.572 and 2.736	Citrate range
15	(2.594 – 2.637)	Unidentified (distribution of singlet lines)
16	2.713	Dimethylamine (most probable)
18	2.883	Trimethylamine
19	3.044 and 3.938	Creatine
20	3.079	Unidentified
21	(3.120,3.147)	Malonate range
22	3.188	Free choline
23	3.228 and 3.241	PC/GPC
24	(3.243 – 3.290) and (3.400 – 3.455)	Taurine
25	3.390	Unidentified
26	3.562	Glycine
27	3.705	Unidentified (broad line at ~3.70 ppm)
28	3.956	Creatine phosphate (most probable)
29	(4.180 – 4.470)	Unidentified (AMP, ADP, ATP, GDP, NADP+)
30	-	*P*

**Figure 3:**
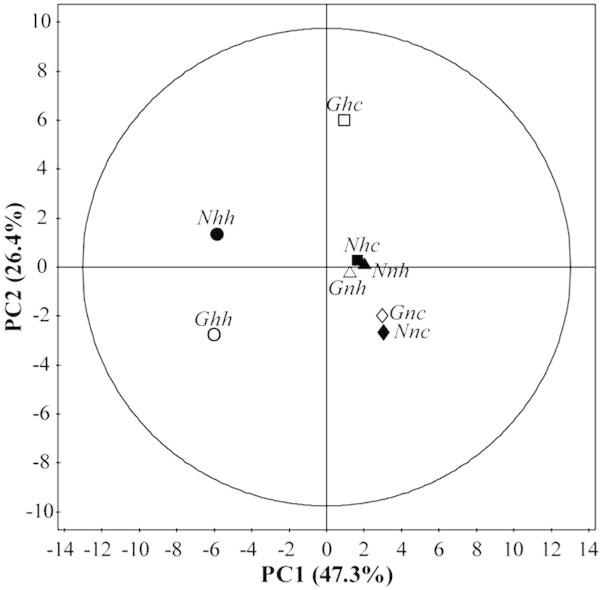
**Principal components analysis (** ***score plot*** **of PC1** ***vs.*** **PC2) from different cell lines (first letter “** ***G*** **” and “** ***N*** **” means** ***G2*** **and** ***N3*** **, respectively) submitted in two different conditions of oxygen tension (second letter “** ***h*** **” and “** ***n*** **” means hypoxia and normoxia, respectively) and glucose levels (third letter “** ***h*** **” and “** ***c*** **” means high and control, respectively), corresponding to 8 mean values of each triplicate.** To facilitate the visualization of results, the open symbols represent the *G2* and solid represent *N3* lines Key: ● *Nhh*, ○ *Ghh*, ■ *Nhc*, □ *Ghc*, ▲ *Nnh*, ∆ *Gnh*, ♦ *Nnc* and ⋄ *Gnc*.

**Figure 4:**
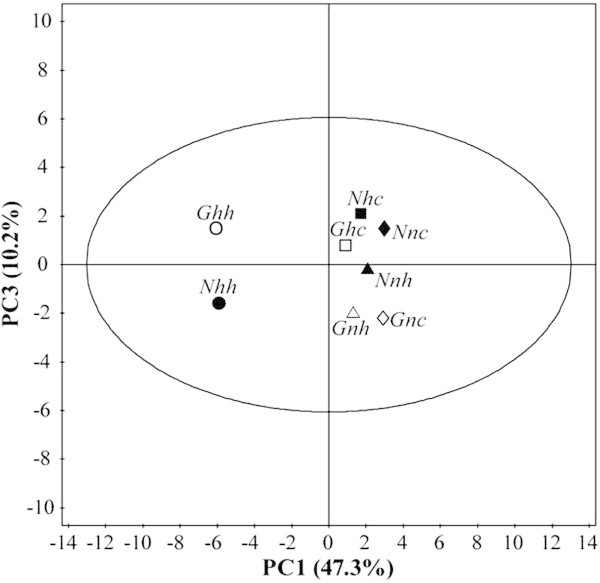
**Principal components analysis (** ***score plot*** **of PC1** ***vs.*** **PC3) from different cell lines (first letter “** ***G*** **” and “** ***N*** **” means** ***G2*** **and** ***N3*** **, respectively) submitted in two different conditions of oxygen tension (second letter “** ***h*** **” and “** ***n*** **” means hypoxia and normoxia, respectively) and glucose levels (third letter “** ***h*** **” and “** ***c*** **” means high and control, respectively), corresponding to 8 mean values of each triplicate.** To facilitate the visualization of results, the open symbols represent the *G2* and solid represent *N3* lines. Key: ● *Nhh*, ○ *Ghh*, ■ *Nhc*, □ *Ghc*, ▲ *Nnh*, ∆ *Gnh*, ♦ *Nnc* and ⋄ *Gnc*.

The integral of different parts of the spectra corresponding to 20 metabolites, 3 fatty acids and 6 unidentified compounds plus *P* (extra absorption line), see Table 1, were used to build the multivariate matrixes of (24 × 30) corresponding to 24 samples *per* 30 integration intervals or in averaging the triplicate of each group the matrix was reduced to a (8 × 30). PC analysis for both matrixes, (24 × 30) and (8 × 30) were performed, Figures 3 and 4 show the results for the reduced matrix, which is almost the same as for the (24 × 30) matrix. The areas were calculated based on trapezoids method in an automatic routine developed in our laboratory using Pascal language.

The first three Principal Components are responsible for approximately 80% of total data variance for PCA. Besides, confidence level on parameters is 95% in PC analysis. To ensure statistical significance for PCA results, ANOVA and Fisher’s test were used to treat each metabolite pointed in multivariate results in order to retain only those comparisons with significance level, α, of 5%. Table 2 resumes these results.

**Table 2:** **Summary of results obtained from ANOVA and Fisher’s test**

***Metabolite***	***l.s.d.***	|Nhh – Ghh|	|Nhh – Ghc|	|Ghh – Ghc|
Lactate	0.03048	0.04277	0.06494	0.02217
Free choline	0.00677	0.00345	0.00945	0.00600
PC/GPC	0.01748	0.00876	0.02713	0.01838
Citrate (P14)	0.01680	0.01035	0.01916	0.02950
P27	0.07465	0.11239	0.01375	0.12614
Creatine	0.01392	0.01980	0.03537	0.05517
Glutamate	0.00257	0.00300	0.00616	0.00916
Succinate	0.00077	0.00003	0.00198	0.00200
Taurine	0.00786	0.01243	0.01271	0.02514
Glycine	0.00324	0.00466	0.00605	0.01071
AMP, ADP, ATP, GDP, NADP^+^	0.01087	0.01098	0.01139	0.02237
Alanine	0.00461	0.00075	0.01343	0.01418

Parameters used were: α = 5% (significance level), *N* = 24 (total of elements in multivariate matrix), *k* = 8 (groups), *n* = 3 (elements by group) and Fisher’s coefficient of 2.12. *Least significant difference* (*l.s.d.*), **|Nhh – Ghh|**, **|Nhh – Ghc|** and **|Ghh – Ghc|** were calculated. These differences were considered statistically significant only when they were greater than *l.s.d.*

Figures 3 and 4 show the plots of PC1 *vs.* PC2 and PC1 *vs.* PC3, which summarize the main results regarding the separation of groups (*score plots*) obtained from PCA. As it can be seem in both figures the separation between groups *Ghh* and *Nhh* are far away from each other which suggests that these two groups have different cellular metabolism.

As it can be seen in Figures 3 and 4*G2* and *N3* cell lines, when both are in hypoxia and higher glucose, are located in different quadrants. In this sense, the PCA results seem to suggest that additionally to the differences between *G2* and *N3* cell lines already mentioned, the oxygen tension and glucose levels are important conditions and somehow they amplify the differences between these cells and therefore should be carefully analyzed. On a biological perspective, galectin-3 may interfere in the metabolism of melanoma cells specifically under such conditions.

In order to discriminate the metabolites responsible for the separation observed in *score plots* of Figures 3 and 4, *loading plots* were analyzed. Table 3 shows the results obtained from the PC1 *vs* PC2 *loading plot*, representing the tendencies of increase and decrease of different metabolites in distinct set of samples. The statistical significance of the changes was tested in Table 2. For example, in third column of Table 3 the metabolites lactate, free choline and PC/GPC are increased in *Nhh* in comparison with *Ghc*.

**Table 3:** **Summary of differences between groups pointed out by the upward arrows mean increases and downward arrows mean decrease**

***Assignments***	***Nhh compared to Ghh***	***Nhh compared to Ghc***	***Ghh compared to Ghc***
Lactate	↑	↑	
Free choline		↑	
PC/GPC		↑	
Citrate (P14)			↑
P27			↑
Creatine		↓	↓
Glutamate		↓	↓
Succinate		↓	↓
Taurine	↑	↓	↓
Glycine		↓	↓
AMP, ADP, ATP, GDP, NADP^+^		↓	↓

Finally, the analysis of this *metabolic chart* with their tendencies of alterations in the samples may provide information regarding the cellular metabolism such as glycolytic pathway, Krebs cycle, mitochondrial regulation, glutaminolysis and PPP pathway.

## Discussion

In this work *Metabolomics* analysis was used in two different cell lines, *G2* (transfected *Tm1* melanoma cells expressing *gal-3*) and *N3* (transfected *Tm1* melanoma cells not expressing *gal-3*), grown at certain conditions of oxygen tension (hypoxia and normoxia) and glucose levels (control or high concentration) in order to identify characteristic metabolic alterations related to *gal-3* expression.

Among the results, two major distinctions regarding the metabolism of observed samples can be established. First, in the PCA charts, the “*hh*” samples are far apart from the rest, implying that hypoxia associated with high glucose levels can have great impacts on cell metabolism, which is rather intuitive. Second, the *Ghh* and the *Nhh* samples have presented wide metabolic differences, demonstrating the impact of *gal-3* expression in the melanoma cell line.

As it can be seen, robustness in the bioenergetic system is maintained in most samples, which are grouped together, but it is violated in the “*hh*” condition. It follows that this medium may have profound effects in cell metabolism. It is worth of note that the glucose diffusion pattern is different from that of oxygen; pO2 declines more rapidly than monosaccharide levels with distance from capillaries (Gatenby and Gillies, 2004). Hence, these conditions may exist inside tumors, which are poorly vascularized.

Doubtless the *Ghh-Nhh* distinction is the richest data obtained. In hypoxia and high glucose conditions, the impact of *gal-3* in bioenergetic reprogramming becomes evident, whatever its precise role may be. Lactate is remarkably increased in *Nhh* cells, probably indicating that under these conditions *gal-3* deficient cells are more glycolytic than *gal-3* expressing cells. However, this does not mean that an *Nhh* tumor will necessarily be more glycolytic than *Ghh* or *Ghc* once the microenvironment cells metabolism is also deregulated during carcinogenesis, a process called reverse Warburg effect (Bonuccelli et al. 2010). Free choline, GPC (glycerophosphocholine) and PC (phosphocholine) are also increased in *Nhh*, being these molecules normally associated with cell membrane biosynthesis (Jansen et al. 2006; Triba et al. 2010), so their concentrations generally reflect an enhanced cellular growth and proliferation typically observed in tumors. In this case, our results show that *Nhh* may be presenting intense cell proliferation rate when compared to *Ghc*. This observation supports the fact that in the intra-cellular environment, *gal-3* gene is involved in regulation of cell proliferation, differentiation and survival through specific interactions with the oncogene K-Ras or with PI3K-Akt signaling pathway.

A behavior was observed for taurine level between two different comparisons among groups. For the case *Nhh* compared to *Ghh*, was observed a decrease of this metabolite for cell lines expressing galectin-3 whereas for *Nhh* compared to *Ghc* was observed an opposite tendency. It is known in literature (Hansen et al. 2006) that taurine is related to the oxidative metabolism which occurs in the mitochondria. Hansen *et al.* (Hansen et al. 2006) demonstrated that high concentration of this metabolite is found in tissues of high oxidative activity, while low concentration is observed in tissues with primarily glycolytic activity. The authors proposed that when the taurine is found inside the mitochondria, it acts to prevent leakage of the Reactive Oxidative Species (ROS) formed in the environment of these organelles, and hence acts as an anti-oxidant agent. Mitochondrial ROS can stabilize HIF, are important signaling molecules and mitogens (Wallace et al. 2010) and also can contribute to neoplastic transformation. It has been observed that in colon cancer cells *gal-3* interacts with F0F1 ATPase, the mitochondrial powerhouse, and may influence cell cycle progression (Kim et al. 2008). These arguments suggest that cellular metabolism may be more pro-oxidative for cell lines which do not expressing *gal-3* gene in hypoxia and high glucose when compared with those that do expressing in same grown conditions. However, when the same comparison between both cell lines is made changing high glucose to control for *G2* lineage, we observed an interesting opposite tendency. This fact, even seeming ambiguous, suggests that only condition “*hh*” may be promoting mitochondrial homeostasis for those cell lines with expressing *gal-3* gene.

All these *gal-3* driven metabolic alterations may have profound impact in cell signaling and consequently in cell growth, division and differentiation. Mitochondrial metabolism changes have great influence in cell signaling, alteration of chromatin methylation and of the epigenome: they can reprogram the nucleus in a process called retrograde signaling (Wallace, 2012). Through specific interactions with HIF or with PI3K pathway, *gal-3* can influence mitochondrial metabolism and hence trigger cancer-related responses.

**Figure 5:**
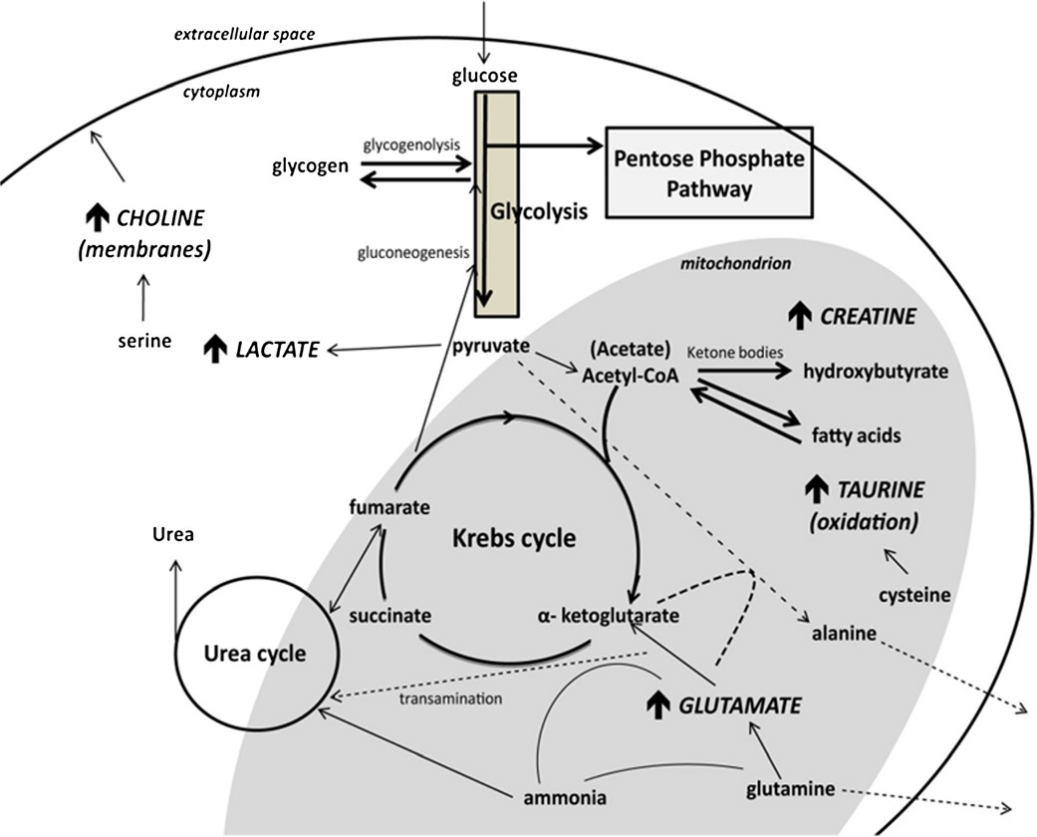
**Schematic overview of changes in cellular metabolism indicated in the two cell lines of melanoma submitted to “** ***hh*** **” condition.** The arrows indicate metabolites which are increased in *Nhh* samples compared to *Ghh*.

Looking at *Ghh-Nhh* peak area comparisons, only few differences could be pointed. Instead, information concerning the metabolic shift emerges when the spectra areas were statistically analyzed and samples were grouped using PCA. Some metabolic differences between *Ghh* and *Nhh* were analyzed and illustrated in Figure 5. In addition to the normal metabolic pathways, the deviations observed in the experiment can be now seen in a contextualized manner.

## Conclusions

*Metabolomics* was an exploratory and non-supervisioned approach through which cells’ metabolic steady states could be compared in presence and absence of *gal-3* expression. Our results showed some changes in concentration of certain metabolites which may be attributed to the impact of galectin-3 in the mitochondrial homeostasis process. Among all metabolites pointed out by multivariate analysis, the most relevant to distinguish between cell lines that expressing *gal-3* gene for those that do not expressing were lactate, free choline, GPC/PC, acetate, 3-hydroxybutyrate and taurine. We propose that the *gal-3* seems to act in the mitochondrial homeostasis only in the specific case where tumorigenic cell are exposed to stress, such as hypoxia condition. A possible explanation for the homeostasis relies on the fact that cells expressing galectin-3 are able to remove from cellular environment those mitochondrias that do not properly metabolize pyruvate received from the glycolytic pathway (“deficient” mitochondria). As discussed, galectin-3 was proven to adapt the cell to a certain environmental pressure and the investigative focus shifted towards these conditions where the samples did not cluster in multivariate analysis. A great spectrum of possibilities emerges to elucidate *gal-3* functionalities.
